# Proteomic analysis and functional validation reveal distinct therapeutic capabilities related to priming of mesenchymal stromal/stem cells with IFN-γ and hypoxia: potential implications for their clinical use

**DOI:** 10.3389/fcell.2024.1385712

**Published:** 2024-05-31

**Authors:** Matteo Calligaris, Giovanni Zito, Rosalia Busà, Matteo Bulati, Gioacchin Iannolo, Alessia Gallo, Anna Paola Carreca, Nicola Cuscino, Salvatore Castelbuono, Claudia Carcione, Claudio Centi, Giandomenico Amico, Alessandro Bertani, Cinzia Maria Chinnici, Pier Giulio Conaldi, Simone Dario Scilabra, Vitale Miceli

**Affiliations:** ^1^ Proteomics Group, Ri.MED Foundation c/o IRCCS ISMETT, Palermo, Italy; ^2^ Research Department, IRCCS ISMETT (Istituto Mediterraneo per i Trapianti e Terapie ad alta Specializzazione), Palermo, Italy; ^3^ Ri.MED Foundation c/o IRCCS ISMETT, Palermo, Italy; ^4^ Thoracic Surgery and Lung Transplantation Unit, IRCCS ISMETT (Istituto Mediterraneo per i Trapianti e Terapie ad alta Specializzazione), Palermo, Italy; ^5^ Regenerative Medicine and Immunotherapy Area, Ri.MED Foundation c/o IRCCS ISMETT, Palermo, Italy

**Keywords:** placenta-derived mesenchymal stromal/stem cells, MSC priming, IFN-γ priming, hypoxia priming, proteomic analysis, MSC therapeutic properties, MSC paracrine effects

## Abstract

Mesenchymal stromal/stem cells (MSCs) are a heterogeneous population of multipotent cells that can be obtained from various tissues, such as dental pulp, adipose tissue, bone marrow and placenta. MSCs have gained importance in the field of regenerative medicine because of their promising role in cell therapy and their regulatory abilities in tissue repair and regeneration. However, a better characterization of these cells and their products is necessary to further potentiate their clinical application. In this study, we used unbiased high-resolution mass spectrometry-based proteomic analysis to investigate the impact of distinct priming strategies, such as hypoxia and IFN-γ treatment, on the composition and therapeutic functionality of the secretome produced by MSCs derived from the amniotic membrane of the human placenta (hAMSCs). Our investigation revealed that both types of priming improved the therapeutic efficacy of hAMSCs, and these improvements were related to the secretion of functional factors present in the conditioned medium (CM) and exosomes (EXOs), which play crucial roles in mediating the paracrine effects of MSCs. In particular, hypoxia was able to induce a pro-angiogenic, innate immune response-activating, and tissue-regenerative hAMSC phenotype, as highlighted by the elevated production of regulatory factors such as VEGFA, PDGFRB, ANGPTL4, ENG, GRO-γ, IL8, and GRO-α. IFN-γ priming, instead, led to an immunosuppressive profile in hAMSCs, as indicated by increased levels of TGFB1, ANXA1, THBS1, HOMER2, GRN, TOLLIP and MCP-1. Functional assays validated the increased angiogenic properties of hypoxic hAMSCs and the enhanced immunosuppressive activity of IFN-γ-treated hAMSCs. This study extends beyond the direct priming effects on hAMSCs, demonstrating that hypoxia and IFN-γ can influence the functional characteristics of hAMSC-derived secretomes, which, in turn, orchestrate the production of functional factors by peripheral blood cells. This research provides valuable insights into the optimization of MSC-based therapies by systematically assessing and comparing the priming type-specific functional features of hAMSCs. These findings highlight new strategies for enhancing the therapeutic efficacy of MSCs, particularly in the context of multifactorial diseases, paving the way for the use of hAMSC-derived products in clinical practice.

## 1 Introduction

Mesenchymal stromal/stem cells (MSCs) are a heterogeneous population of multipotent cells that can be isolated from various adult or neonatal tissues, including dental pulp, adipose tissue, bone marrow, umbilical cord and placenta, among others (Parolini et al., 2008; Hass et al., 2011; Burja et al., 2020). These cells play a physiological role in tissue repair and regeneration due to their inherent regulatory abilities (Jackson et al., 2012; Morrison and Scadden, 2014; Wosczyna et al., 2019; Miceli et al., 2020; Schmelzer et al., 2020; Lo Nigro et al., 2021), and therefore have been widely explored as promising candidates for therapeutic applications within the field of regenerative medicine (Iijima et al., 2018; Chinnici et al., 2021; Cittadini et al., 2022; Margiana et al., 2022; Miceli and Bertani, 2022; Miceli et al., 2023a; Miceli et al., 2023b; Russo et al., 2023b). Over the years, scientific evidence has highlighted that the therapeutic effects of MSCs are, at least in part, mediated by the secretion of paracrine functional factors and/or biovesicles, including cytokines, chemokines, growth factors, and extracellular vesicles (EVs) such as exosomes (EXOs) (Caplan and Dennis, 2006; Miceli et al., 2021b; Papait et al., 2022; Miceli et al., 2023b; Cui et al., 2023). Consequently, considering the role of paracrine activity in the beneficial effects of MSCs, there is growing interest in elucidating the molecular mechanisms underlying MSC secretion, as this process is crucial for their therapeutic efficacy. Notably, EXOs represent a fundamental functional component of the secretome that is responsible for mediating the paracrine effects of MSCs (Takeuchi et al., 2021; Alberti et al., 2022; Russo et al., 2023a; Bulati et al., 2023). In this case, it is very important to analyze the EXO fraction to characterize its contents and establish its functional role in the context of the MSC secretome.

The examination of MSC secretome features has become fundamental, especially considering the variability in therapeutic outcomes observed in clinical trials where MSCs were used to treat a wide range of diseases, including orthopedic, neurodegenerative, cardiovascular, lung, liver, and kidney diseases (Chen et al., 2023) (as evidenced by 1613 registered clinical trials on clinicaltrials.gov; 03 February 2024). In fact, many studies have previously shown that although MSC-based therapies have demonstrated good safety and tolerability profiles (Wang Y. et al., 2021; Uccelli et al., 2021), their effectiveness varies significantly, often resulting in minimal or no discernible effects (Squillaro et al., 2016; Lukomska et al., 2019; Fricova et al., 2020; Zhou et al., 2021). The different therapeutic outcomes have been linked to the high degree of heterogeneity that characterizes MSCs. In recent years, many findings have associated this observed MSC heterogeneity with both intrinsic biological aspects, such as differences in tissue origin and donor-to-donor variation in MSC function (McLeod and Mauck, 2017; Zha et al., 2021), and technical aspects, such as differences in the harvesting and culturing laboratory methods required for MSC expansion before clinical application (Stroncek et al., 2020; Wang Y. H. et al., 2021). Both cell sources and surface markers have proven to be unreliable as indicators of MSC therapeutic success (Robey, 2017). In this regard, however, the International Society for Cell and Gene Therapy (ISCT) recommend that the definition of MSCs be integrated with their tissue origin to underscore the tissue-specific properties of MSCs, which might be linked to expected therapeutic actions (Viswanathan et al., 2019). Several studies have suggested that MSCs isolated from different tissues exhibit distinct phenotypes and functional properties (Melief et al., 2013; Phinney and Sensebe, 2013). This heterogeneity has led some research groups to carefully characterize the MSC secretome in terms of trophic (Hofer and Tuan, 2016), immunomodulatory (Munoz-Perez et al., 2021), and pro-angiogenic factors (Maacha et al., 2020). In particular, quantitative proteomic analyses have revealed functional disparities between the secretomes of MSCs derived from fetal and adult skin (Gaetani et al., 2018). Additionally, using liquid chromatography-tandem mass spectrometry, variable angiogenic potential has been demonstrated between the secretomes of MSCs derived from adipose tissue, bone marrow, and Wharton’s jelly (Kehl et al., 2019). These recent findings reveal that MSCs isolated from diverse tissue sources possess distinct proteomic and functional traits and underscore the challenge of establishing consistent conclusions regarding the true therapeutic efficacy of MSCs.

To address these issues, many studies have focused on the concept of preconditioning MSCs (MSC priming) before their clinical use as a possible strategy to enhance and modulate the beneficial therapeutic properties of MSCs. In this regard, different stimuli and culture conditions, such as hypoxia exposure, cytokine treatments and 3D culture conditions have been used to direct MSCs toward specific immunomodulatory or trophic effects, thereby augmenting their regenerative potential (Ferreira et al., 2018; Miceli et al., 2019; Gallo et al., 2022). Several studies have shown that specific priming strategies at different stages of MSC production can modify the MSC secretome (Miceli et al., 2020; Miceli et al., 2021a; Zito et al., 2022; Bulati et al., 2023; Chouaib et al., 2023), emphasizing the potential role of the preconditioning strategy in the standardization of this approach (Dunn et al., 2021; Miceli et al., 2023b; Chouaib et al., 2023). For instance, IFN-γ has been employed as a crucial activator of MSC-mediated immunosuppression by upregulating the production of indoleamine 2,3-dioxygenase 1 (IDO), prostaglandin E synthase 2 (PGE2), interleukin 10 (IL10), and CCL4 (MIP1B) (Kim et al., 2018; Bulati et al., 2020). Preclinical studies have also demonstrated that IFN-γ-primed MSCs exhibit greater efficacy than naïve cells in various immune-related disease models (Duijvestein et al., 2011; Kanai et al., 2021). As anticipated, the composition of the MSC secretome can also be modulated by preconditioning MSCs under hypoxia. In this regard, in both *in vitro* and *in vivo* models, it has been revealed that when MSCs are exposed to temporary hypoxia, mimicking the stem cell niche microenvironment, they undergo genetic transcription changes that lead to an increase in cytoprotective and regenerative abilities, along with improved angiogenic properties (Leroux et al., 2010; Wei et al., 2012; Hu et al., 2016).

In this study, using MSCs derived from the amniotic membrane of the human placenta (hAMSCs), we aim to characterize the hAMSC secretome by evaluating both secreted and exosomal proteins. Our goals are to comprehensively investigate the impact of IFN-γ or hypoxia priming on hAMSCs and to systematically assess and compare priming type-specific functional features.

## 2 Materials and methods

### 2.1 Isolation, culture and phenotypic characterization of amnion-derived mesenchymal stromal/stem cells

To obtain MSCs, written informed consent was obtained from each donor, and the procedure was approved by IRCCS ISMETT’s Institutional Research Review Board (IRRB, code: IRRB/39/20). MSCs were isolated from the amniotic membrane of the human term placenta (38–40 weeks of gestation) of healthy donors within 6 h of birth. The amnion was mechanically separated from the chorion and washed several times in phosphate-buffered saline (PBS). The amniotic membrane was then cut into pieces of 3 × 3 cm^2^ and each piece was decontaminated via incubation in PBS supplemented with 2.5% iodopovidone (Esoform, Italy) for a few seconds, followed by incubation in PBS containing 500 U/mL penicillin, 500 mg/mL streptomycin, 12.5 mg/mL amphotericin B, and 1.87 mg/mL cefamezin (Pfizer, Italy) for 3 min and subsequent incubation in PBS containing 100 U/mL penicillin and 100 mg/mL streptomycin for 5 min. Decontaminated fragments were treated for 9 min at 37°C in hanks’ balanced salt solution (HBSS) (Lonza, CH) containing 2.5 U/mL dispase (Corning, NY, United States). To neutralize the dispase, the fragments were incubated for 5 min at room temperature in roswell park memorial institute (RPMI) 1640 complete medium (Invitrogen, United States) supplemented with 10% fetal bovine serum (FBS) (HyClone, United States) and then digested with 0.94 mg/mL collagenase A (Roche, Germany) and 20 mg/mL DNase (Roche, Germany) for 2.5 h at 37°C. The digest was successively filtered through 100 μm and 70 μm cell strainers (BD Falcon, United States), pelleted by centrifugation at 150–300 g for 10 min, and resuspended in RPMI 1640 complete medium (Invitrogen, United States) supplemented with 10% FBS for cell counting. The cells obtained were cultured in polystyrene culture dishes (Corning, NY, United States) at 37°C and 5% CO_2_ in Chang medium (Irvine Scientific, United States) for the first step and expansion. For phenotypic characterization, hAMSCs were first washed twice with FACS buffer (PBS containing 0.3% BSA and 0.1% NaN_3_) and then incubated on ice for 30 min with specific antibodies against CD90, CD73, CD13, CD45, and HLA-DR (BD Biosciences, United States). Finally. the cells were washed twice with FACS buffer and analyzed using the FACSCelesta™ cytometer (BD Life Sciences, United Kingdom).

### 2.2 Priming and conditioned medium preparation

For conditioned medium (CM) collection from cultures with or without priming, hAMSCs at the second step were cultured in Chang medium until 90% confluence. Then the medium was replaced with serum-free dulbecco’s modified eagle medium (DMEM) medium supplemented with or without 200 IU/mL IFN-γ (Human IFN-g1b premium grade, Miltenyi Biotec, Germany) and the cells were grown at 37°C, 20% O_2_ and 5% CO_2_. For CM collection from hypoxic priming, the cells were cultured with serum-free DMEM medium at 37°C, 1% O_2_ and 5% CO_2_. The supernatants from all the cultures were harvested after 48 h and frozen at −80°C until use.

### 2.3 Isolation and characterization of exosomes

**FIGURE 1:**
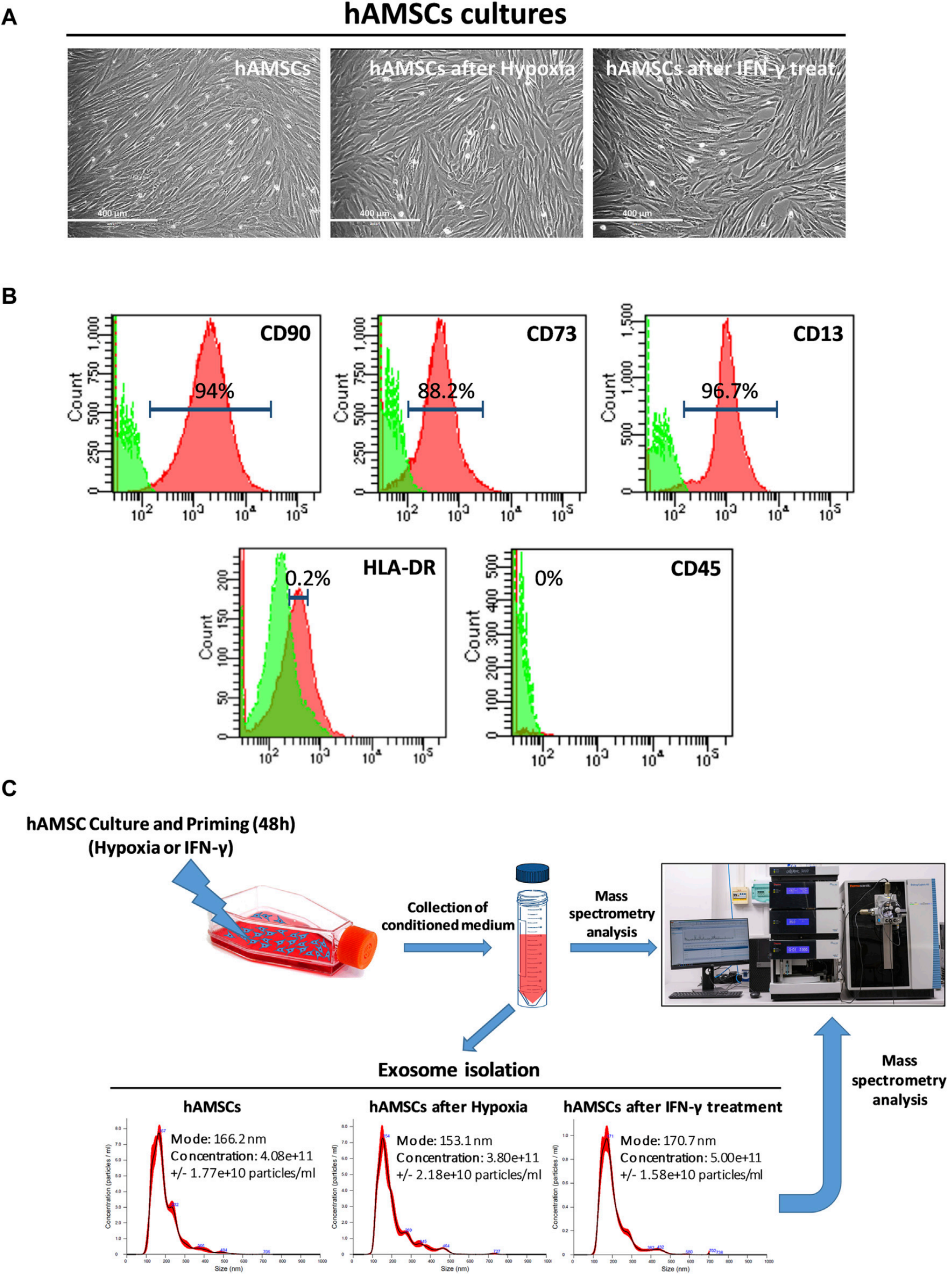
Human amnion-derived mesenchymal stromal/stem cells (hAMSCs) were grown as monolayers with or without priming. **(A)** Representative DIC images of hAMSCs grown as monolayers without priming (hAMSCs), cultured under hypoxic conditions (hAMSCs after hypoxia), or treated with IFN-γ (hAMSCs after IFN-γ treatment). **(B)** Representative images of flow cytometry analysis for quantification of hAMSCs at step 2 for both positive (CD90, CD73 and CD13) and negative surface markers (HLA-DR and CD45). Green represents the isotype control, and red represents stained cells. **(C)** Experimental plan and exosome characterization (size and concentration). DIC, differential interference contrast.

EXOs were isolated from each primed and unprimed CM through ultracentrifugation. The CM was centrifuged at 300 *g* for 10 min to remove the debris. To further remove both cells and debris, the CM was centrifuged for 20 min at 16500 × g and then ultracentrifuged at 120000 × g for 90 min at 4°C to pellet the EXOs. The total protein content of the EXO preparations was determined using the Micro BCA Protein Assay Reagent Kit following the manufacturer’s instructions (Thermo Scientific, United States). To characterize the EXOs, their size, distribution, and concentration (Figure 1C) were determined via nanoparticle tracking analysis (NTA) in a NanoSight NS3000 (Malvern Instruments Ltd., United Kingdom). The samples were diluted 1:500 with PBS to reach an optimal concentration for instrument linearity. Readings were taken in quintuplicate of 60 s at 25 frames per second, and the data obtained were then analyzed with NTA software version 3.1 (Build 3.1.54, Analytik Ltd, United Kingdom).

### 2.4 Mass spectrometry analysis

2 mL of conditioned medium or 10 µg of isolated exosomes from differentially stimulated MSCs underwent filter aided sample preparation (FASP) with 10 kDa Vivacon 500 spin filters (Wisniewski et al., 2009). Briefly, proteins were reduced with 20 mM dithiothreitol for 30 min at 37°C and free cysteine residues were alkylated with 50 mM iodoacetamide for 5 min at 37°C in the dark in UA (100 mM Tris/HCl 8 M urea pH 8.5) (Sigma Aldrich). After six washing steps with UB (100 mM Tris/HCl 8 M urea pH 8), proteins were digested with 0.3 µg LysC (Promega) in UC (25 mM Tris/HCl 2 M urea pH 8) for 16 h at 37°C followed by a second digestion step with 0.15 µg trypsin in 50 mM ammonium bicarbonate (Promega) for 4 h at 37°C as previously described (Saveliev et al., 2013). The peptides were eluted into collection tubes and acidified with formic acid (Sigma Aldrich) at a final concentration of 0.1%. Afterward, proteolytic peptides were desalted by stop-and-go extraction (STAGE) with self-packed C18 tips (Empore) (Rappsilber et al., 2003). Peptides were eluted using 60% acetonitrile (Sigma Aldrich) and 0.1% formic acid. After vacuum centrifugation, peptides were concentrated and dissolved in 20 µL 0.1% formic acid. At this point, peptide concentrations were analyzed by a Nanodrop 2000 (Thermo Scientific). A total amount of 1.2 μg was loaded per sample onto a Dionex UltiMate 3000 RSLCnano LC system (Thermo Scientific) which was coupled online via a Nanospray Flex Ion Source (Thermo Scientific) to a Q Exactive mass spectrometer (Thermo Scientific). Peptides were separated on an Acclaim PepMap C18 column (50 cm × 75 µm ID, Thermo Scientific) with 250 Nl/min flow using a binary gradient of water (A) and acetonitrile (B) supplemented with 0.1% formic acid (2% B 0 min, 5% B 5 min, 25% B 185 min, 35% B 230 min, 60% B 250 min, 95% B 255 min, 95% B 265 min, 2% B 265 min, 2% B 350 min). Data-dependent acquisition (DDA) was used for label-free quantification (LFQ). Full MS scans were acquired at a resolution of 70,000 (m/z range: 300–1400; automatic gain control (AGC) target: 1E+6; max injection time 50 ms). The DDA was used on the 10 most intense peptide ions per full MS scan for peptide fragmentation (resolution: 17,500; isolation width: 2 m/z; AGC target: 10^5^; normalized collision energy (NCE): 25%, max injection time: 120 ms). A dynamic exclusion of 120 s was used for peptide fragmentation.

The raw data were analyzed with the software MaxQuant, version 2.0.1.0 (maxquant.org, Max Planck Institute, Munich). The MS data were searched against a reviewed canonical FASTA database of *Homo sapiens* including isoforms from UniProt (download: November the 5th 2020). Trypsin/P was defined as a protease. Two missed cleavages were allowed for the database search. The option in the first search was used to recalibrate the peptide masses within a window of 20 ppm. For the main search peptide and peptide fragment mass tolerances were set to 4.5 and 20 ppm, respectively. Carbamidomethylation of cysteine was defined as static modification. Protein N-terminal acetylation as well as oxidation of methionine were set as variable modifications. The false discovery rate for both peptides and proteins was adjusted to less than 1%. The “match between runs” option was enabled. LFQ of proteins required at least one ratio count of unique peptides. Unique and razor peptides were used for quantification. Data normalization was enabled. The protein LFQ reports from MaxQuant were further processed in Perseus (Tyanova et al., 2016).

### 2.5 Cluster and gene ontology (GO) analysis

Hierarchical cluster analysis of protein expression (expressed as the z-score) was used to group treatments with similar expression patterns. Protein expression data were grouped using a hierarchical clustering algorithm in the Cluster 3.0 program. A heatmap was generated using the Java TreeView program. To find GO terms enriched in the significantly deregulated proteins, we analyzed our data with the STRING web tool (Szklarczyk et al., 2019).

### 2.6 Endothelial cell cultures and tube formation assay

Human umbilical vein endothelial cells (HUVECs) were obtained from ATCC (United States). HUVECs were maintained in an endothelial cell basal medium (Lonza/Clonetics Corporation, United States) supplemented with a BulletKit (EBM-2) (Lonza/Clonetics Corporation, United States) in a culture flask coated with 0.1% gelatin (STEMCELL Technologies, United States) and maintained at 37°C with 5% CO_2_.

A tubulogenesis assay was performed with basement membrane extract (BME) type 2 (AMSBIO, United Kingdom). HUVECs were dispensed at 1 × 10^4^ cells/well (96-well microplates, Nunc, Germany) on top of the BME in serum-free DMEM (negative control), serum-free DMEM supplemented with 30 or 60 μg/mL EXOs derived from primed MSCs, or each conditioned medium (with or without EXOs) (100 μL). Following incubation at 37°C and 5% CO_2_ for 6 h, the cells were visualized and images were taken using an EVOS™ FL digital inverted fluorescence microscope (Fisher Scientific, United Kingdom). The number of nodes, branches and meshes, along with the length of the master segments, branches and tubes were measured with ImageJ software (National Institutes of Health, USA). For statistical significance, six images/replicate were analyzed and quantified (*n* = 3).

### 2.7 Endothelial migration assay (xCELLigence)

Real-time monitoring of HUVECs was performed with an xCELLigence system (ACEA, United States) using CIM-Plate 16. The upper chamber was seeded with 30,000 HUVECs in serum-free DMEM medium. When endothelial cells migrated through the membrane into the bottom chamber in response to attractants (160 μL of complete DMEM as a positive control; 160 μL of serum-free DMEM without treatments (not treated, NT), or conditioned medium by each treatment, or serum-free DMEM with EXOs), they adhered to the electronic sensors resulting in increased impedance. The cell index (CI) values reflecting impedance changes were automatically recorded every 15 min. All culture conditions were carried out in quadruplicate and the analysis was performed with RTCA Software 1.2 from the xCELLigence system.

### 2.8 Neutrophil isolation and cell migration assay (xCELLigence)

Neutrophils were magnetically isolated from the whole blood of 3 healthy donors (two males and one female aged between 36 and 50 years) contained in a Vacutainer K2-EDTA tube (Becton Dickinson, San Jose, CA, United States) using StraightFrom Whole Blood CD66b MicroBeads (Miltenyi Biotec, Germany). The magnetically retained CD66b^+^ neutrophils were used for cell migration assay performed with the xCELLigence system (ACEA, United States). The upper chamber was seeded with 100,000 neutrophils in DMEM serum-free medium. When neutrophil cells migrated into the bottom chamber in response to attractants (160 μL of serum-free DMEM without treatments, NT, or conditioned medium by each treatment, or serum-free DMEM with EXOs), they adhered to the electronic sensors resulting in increased impedance. Cell index was registered every 15 min reflecting impedance changes. We used N-Formyl-Met-Leu-Phe (N-fMLP) (Sigma-Aldrich, Germany) at 1 µM as a positive control for neutrophil migration. Each culture condition was carried out in quadruplicate and the analysis was performed by RTCA Software 1.2 from the xCELLigence system.

### 2.9 Phagocytosis assay

The phagocytosis assay was performed by exposing heparinized blood samples (three different donors, two males and one female aged between 36 and 50 years) to pHrodo Green *E. coli* BioParticles (catalog no. P35366, Invitrogen) according to the manufacturer’s instructions. The bioparticles were reconstituted in uptake buffer (20 mM HEPES in HBSS, pH 7.4) to a concentration of 1 mg/mL. The blood was pre-incubated for 1 h with 30 µL of each CM with or without EXOs (NO PRIM CM, IFN-γ CM and HYP CM) or two concentrations (30 or 60 μg/mL) of the three different EXOs (NO PRIM, IFN-γ, HYP) followed by 2 h of incubation with pHrodo *E. coli* BioParticles. At the end of the incubation time, the blood samples were lysed at room temperature for 10 min, washed and stained with CD45 APC-Conjugated antibody (Miltenyi Biotec) and 7AAD (Miltenyi Biotec), and immediately acquired by a FACSCelesta™ cytometer and analyzed with FlowJo™ v10.8.1 software (BD Life Sciences, United Kingdom).

### 2.10 Simultaneous quantification of secreted cytokines

Venous blood (three different donors, two males and one female aged between 36 and 50 years) was collected in K3EDTA tubes (Greiner Bio-One GmbH, Austria) and diluted 4-fold in RPMI 1640 medium supplemented with 1% penicillin/streptomycin, 10 mM HEPES (Euroclone, Pero, Italy), and 1 mM L-glutamine (Lonza Group Ltd, Switzerland). The blood was pre-incubated for 1 h with 30 µL of each CM with or without EXOs (NO PRIM CM, IFN-γ CM and HYP CM) or 30 μg/mL of three different exosomes (EXOs, IFN-γ EXOs, HYP EXOs) followed by 4 and 24 h of stimulation with *E. coli* LPS 1 μg/mL (*E. coli* O127:B8 Sigma-Aldrich). The samples were incubated at 37°C and 5% CO_2_. After the incubation time with LPS, the levels of selected functional factors were assessed in the supernatants using Luminex magnetic bead technology with the ProcartaPlex Multiplex Immunoassay according to the manufacturer’s instructions (Affymetrix, Austria).

### 2.11 Statistics

All the results were expressed as the mean ± standard deviation (SD). Statistical analysis was performed using GraphPad Prism 6.0 (GraphPad Software, United States). For statistical comparison, the one-way ANOVA with Tukey multiple comparison test was used. *p*-values <0.05 were considered to indicate statistical significance (*p < 0.05, **p < 0.01, ***p < 0.001, ****p < 0.0001).

## 3 Results

### 3.1 Isolation, cultivation, and characterization of hAMSCs and collection of both CM and EXOs

MSCs from human amniotic membranes were isolated from the placentas of distinct donors (*n* = 4) and cultured in the appropriate Chang medium. Primary cultures of hAMSCs were expanded *in vitro* until step 2 and then grown in conventional culture (hAMSCs), in hypoxia (hAMSCs after hypoxic treatment), or in the presence of IFN-γ (hAMSCs after IFN-γ treatment). All the cultures exhibited an elongated and fibroblastic-like morphology typical of MSCs (Figure 1A). The hAMSCs were analyzed by flow cytometry and were found to be positive for CD90 (94%), CD73 (88.2%), and CD13 (96.7%), and negative for HLA-DR (0.2%) and CD45 (0%) (Figure 1B). After hAMSC priming, no differences in mortality were found between the treatments (data not shown). As depicted in Figure 1C, following 48 h of culture, we harvested the CM generated from the hAMSCs and isolated EXOs from each CM. We obtained EXOs with an average diameter of 163 nm from hAMSCs and hypoxia-treated hAMSCs at similar concentrations (4.08 × 10^11^ and 3.80 × 10^11^ particles/mL, respectively), while a higher EXO concentration was produced by IFN-γ-treated hAMSCs (5.00 × 10^11^ particles/mL). We also confirmed differences in EXO concentrations through the analysis of EXO protein levels, which were similar in EXOs produced from hAMSCs and hypoxia-treated hAMSCs (18.545 μg/mL and 17.272 μg/mL, respectively) and greater in EXOs produced from IFN-γ-treated hAMSCs (22.727 μg/mL).

### 3.2 Proteomic analysis of hAMSC CM and EXOs reveals distinct patterns of protein secretion associated with specific priming methods impacting different biological processes

We used unbiased high-resolution mass spectrometry-based proteomics to compare the proteome of both CM and EXOs after hypoxic or IFN-γ priming of hAMSCs. A total of 1476 and 1441 different proteins were identified in CM and EXOs, respectively (for the list of all proteins, see Supplementary Table S1 for CM and S2 for EXOs). Only the proteins that were detected in CM samples from at least three out of four donors or in EXO samples from at least two out of three donors were taken into consideration.

**FIGURE 2:**
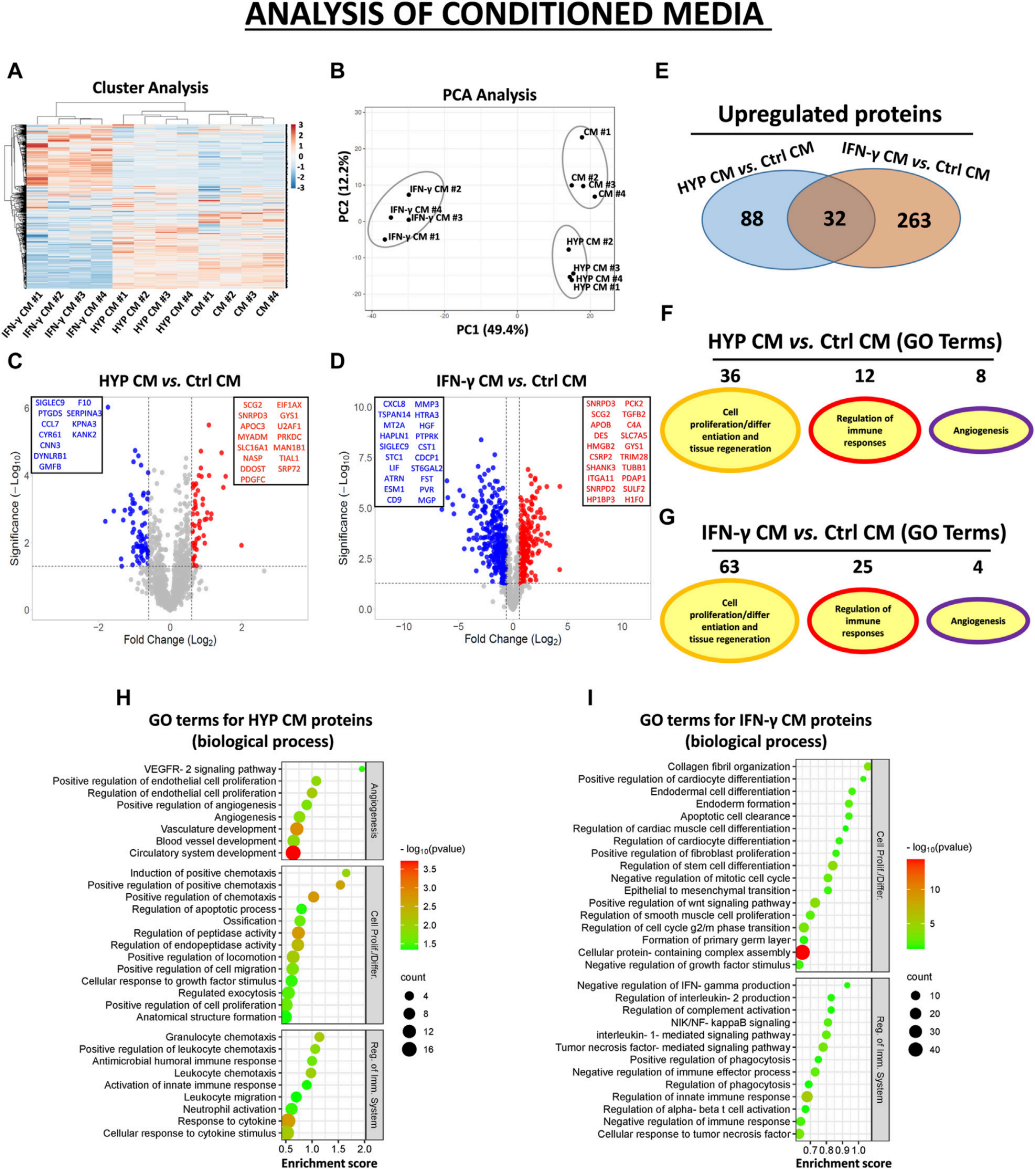
Protein secretion profiles in conditioned medium (CM) derived from unprimed hAMSCs (ctrl) and primed hAMSCs under hypoxia (HYP) or IFN-γ. **(A)** Secretion clusters (z-scores) of both up- and downregulated proteins in CM derived from hAMSCs (CM), hypoxic hAMSCs (HYP CM) and IFN-γ-treated hAMSCs (IFN-γ CM). **(B)** Principal component analysis (PCA). **(C)** Volcano plot analysis (fold change >1.5 and *p* < 0.05) of secreted proteins in HYP CM vs. ctrl CM and **(D)** IFN-γ CM vs. ctrl CM. **(E)** Venn diagram showing the number of upregulated proteins in HYP CM and IFN-γ CM. **(F)** Number of GO-enriched terms associated with upregulated HYP CM and **(G)** IFN-γ CM proteins grouped by category. **(H)** GO enrichment terms of HYP CM- and **(I)** IFN-γ CM-upregulated proteins; partial list of the 30 most significantly enriched terms.

**FIGURE 3:**
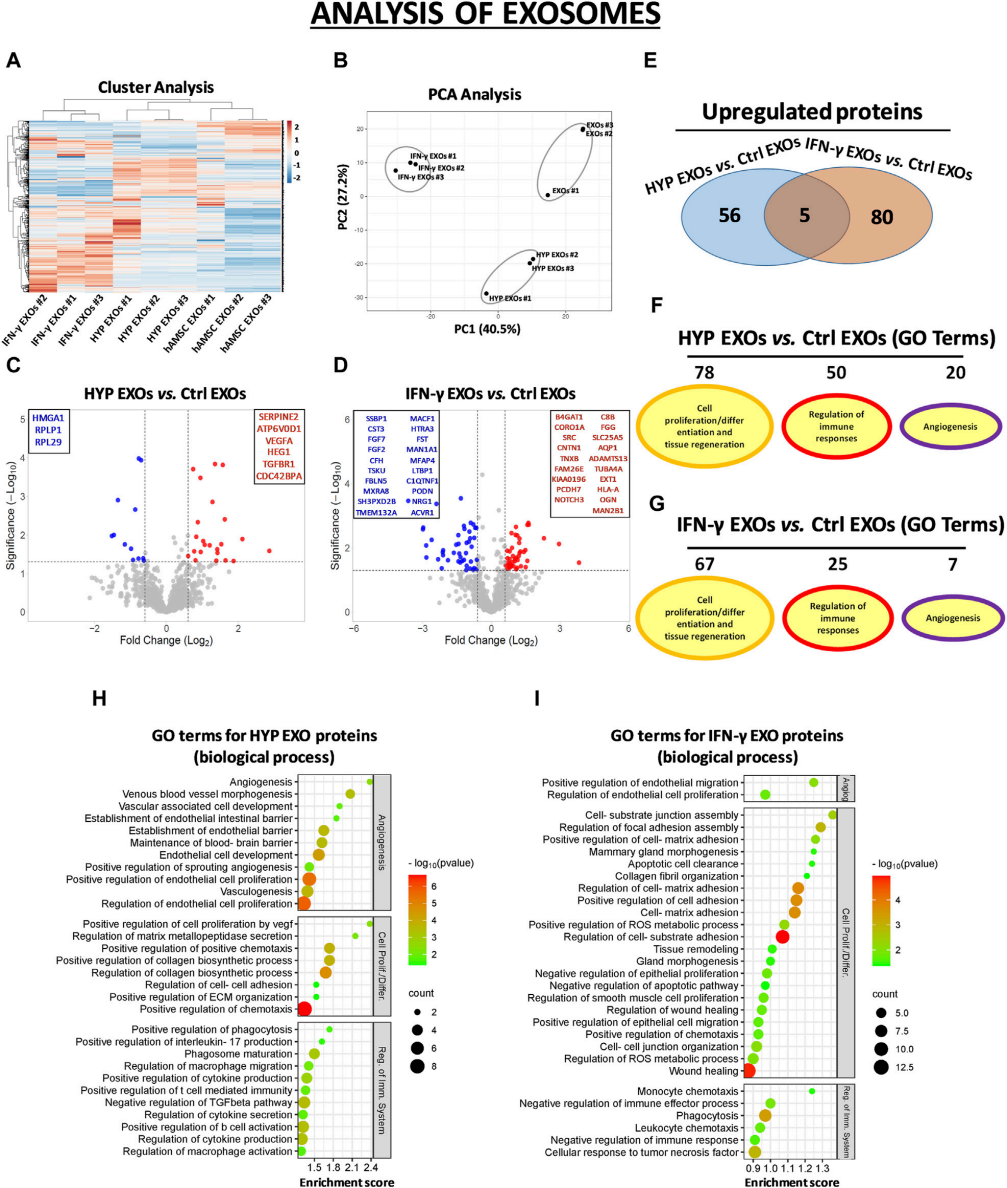
Protein secretion profiles of exosomes (EXOs) derived from unprimed hAMSC ctrl and primed hAMSCs under hypoxia (HYP) or IFN-γ. **(A)** Secretion clusters (z-scores) of both up- and downregulated proteins in EXOs derived from hAMSCs (hAMSC EXOs), hypoxic hAMSCs (HYP EXOs) and IFN-γ-treated hAMSCs (IFN-γ EXOs). **(B)** Principal component analysis (PCA). **(C)** Volcano plot analysis (fold change >1.5 and *p* < 0.05) of secreted protein in HYP EXOs vs. ctrl EXOs and **(D)** IFN-γ EXOs vs. ctrl EXOs. **(E)** Venn diagram showing the number of upregulated proteins in HYP EXOs and IFN-γ EXOs. **(F)** Number of GO-enriched terms associated with upregulated HYP EXO and **(G)** IFN-γ EXO proteins grouped by category. **(H)** GO enrichment terms of HYP EXO- and **(I)** IFN-γ EXO-upregulated proteins; partial list of the 30 most significantly enriched terms.

In the CM samples, the normalized heatmap revealed a distinct proteomic profile between primed and unprimed hAMSCs, as well as between hypoxic or IFN-γ priming (Figure 2A). These data were confirmed by principal component analysis (PCA) (Figure 2B). We statistically analyzed deregulated proteins across groups by volcano plot analysis (fold change >1.5 and *p*-value <0.05) and detected significant changes in protein secretion under the different priming conditions. Specifically, compared to those in the conventional hAMSC CM (control CM), 88 proteins were significantly upregulated and 69 were lost in the hypoxic hAMSC CM (HYP CM) (Figures 2C–E), while 263 proteins were upregulated and 477 were downregulated in the IFN-γ CM (Figures 2D,E). We then performed a GO analysis to investigate whether deregulated proteins affect biological processes related to tissue repair/regeneration, immune system regulation and angiogenesis, which are of particular interest in the field of regenerative medicine. Notably, compared to IFN-γ priming, hypoxic priming appears to be more effective at inducing the overexpression of CM functional proteins that regulate angiogenic pathways, while more GO terms related to tissue repair/regeneration and immune system regulation were targeted by overexpressed proteins detected in IFN-γ CM (Figures 2F,G). A list of the top 30 GO terms related to HYP- and IFN-γ-upregulated proteins is shown for CM in Figures 2H,I, and the complete list of GO-enriched terms is displayed in Supplementary Table S3. A similar trend was observed for the EXO samples. In particular, both heatmap and PCA analyses revealed differences in proteomic profiles between EXOs produced from unprimed hAMSCs, hypoxic hAMSCs and IFN-γ-treated hAMSCs (Figures 3A,B). Regarding the protein content in the EXO samples, compared with unprimed hAMSC EXOs, hypoxic priming (HYP EXOs) resulted in the upregulation of 56 proteins and the disappearance of 15 proteins (Figures 3C–E). In addition, IFN-γ treatment (IFN-γ EXOs) led to the upregulation of 80 proteins and the downregulation of 66 proteins (Figures 3D,E). In contrast to the upregulated proteins in CM, when we analyzed overexpressed proteins in EXOs, compared to those in IFN-γ EXOs, we observed that the overproduced factors obtained in hypoxic priming (HYP EXOs) targeted more GO terms related to tissue repair/regeneration, the immune system and angiogenesis regulation (Figures 3F,G). The top 30 GO terms related to proteins contained in HYP EXOs and IFN-γ EXOs are shown in Figures 3H,I, and the complete list is presented in Supplementary Table S4. A total of 32 (Figure 2E) and 5 (Figure 3E) proteins exhibited increased expression with hypoxic and IFN-γ priming in CM and EXOs, respectively.

### 3.3 Conditioned medium and exosomes produced through hypoxic priming contain the highest angiogenic proteome

**FIGURE 4:**
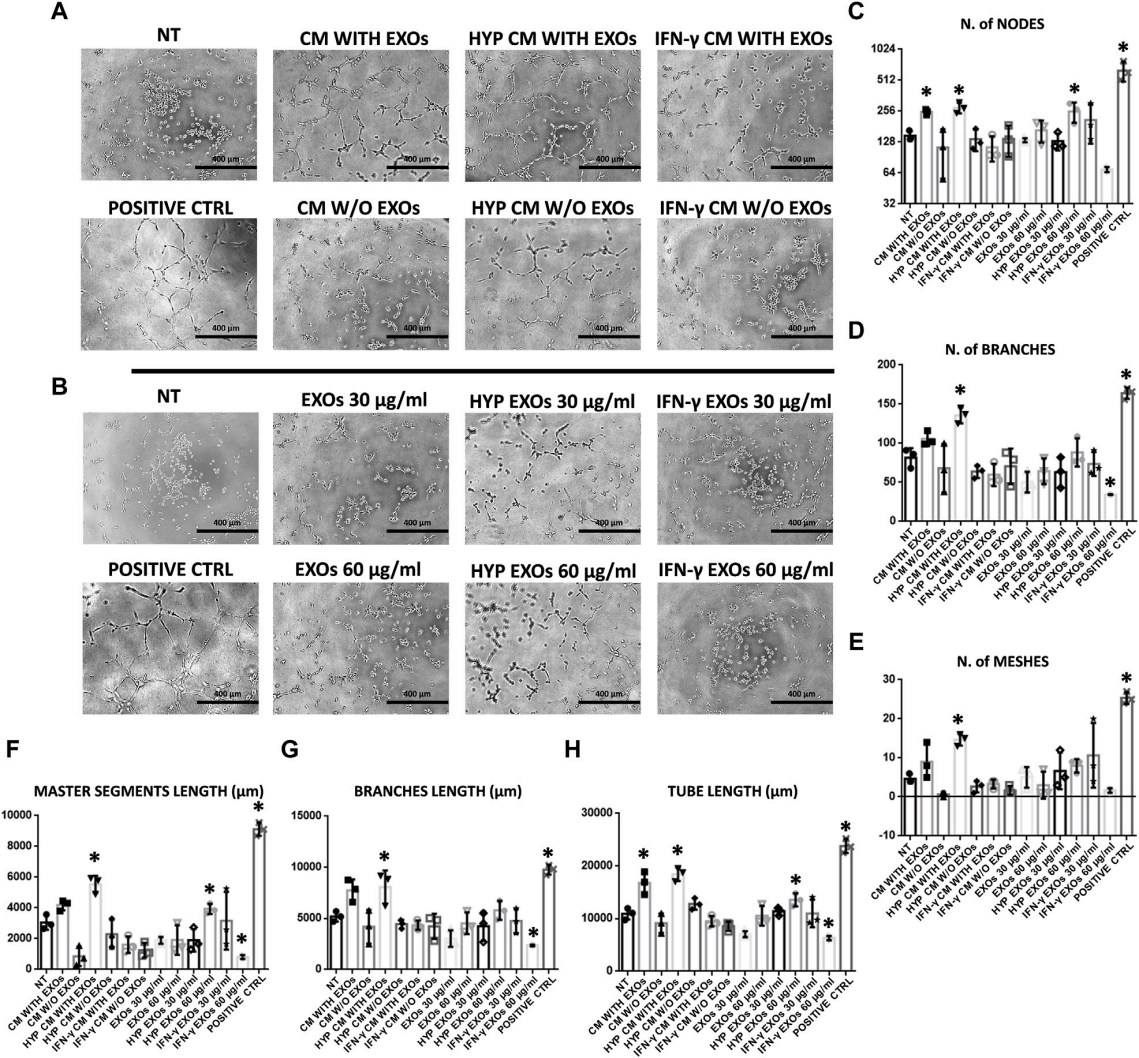
HUVEC capillary-like formation assay. **(A)** Representative images of HUVECs on BME treated with conditioned medium (CM) or **(B)** exosomes (EXOs). **(C–H)**- Graphs represent a quantitative analysis of the **(C)** number of nodes, **(D)** number of branches, **(E)** number of meshes, **(F)** master segment length, **(G)** branch length, and **(H)** tube length. Untreated serum-free DMEM (NT); Untreated DMEM with serum (positive ctrl); Serum-free DMEM conditioned by hAMSCs (CM with EXOs); Serum-free DMEM conditioned by hAMSCs depleted of EXOs (CM w/o EXOs); Serum-free DMEM conditioned by hypoxic hAMSCs (HYP CM with EXOs); Serum-free DMEM conditioned by hypoxic hAMSCs depleted of EXOs (HYP CM w/o EXOs); Serum-free DMEM conditioned by IFN-γ-treated hAMSCs (IFN-γ CM with EXOs); Serum-free DMEM conditioned by IFN-γ-treated hAMSCs depleted of EXOs (IFN-γ CM w/o EXOs); 30 μg/mL EXOs secreted by hAMSCs (EXOs 30 μg/mL); 60 μg/mL EXOs secreted by hAMSCs (EXOs 60 μg/mL); 30 μg/mL EXOs secreted by hypoxic hAMSCs (HYP EXOs 30 μg/mL); 60 μg/mL EXOs secreted by hypoxic hAMSCs (HYP EXOs 60 μg/mL); 30 μg/mL EXOs secreted by IFN-γ-treated hAMSCs (IFN-γ EXOs 30 μg/mL); 60 μg/mL EXOs secreted by IFN-γ-treated hAMSCs (IFN-γ EXOs 60 μg/mL). Data are presented as the means ± SD of triplicate in two independent experiments. ∗*p* < 0.05 vs. NT.

**FIGURE 5:**
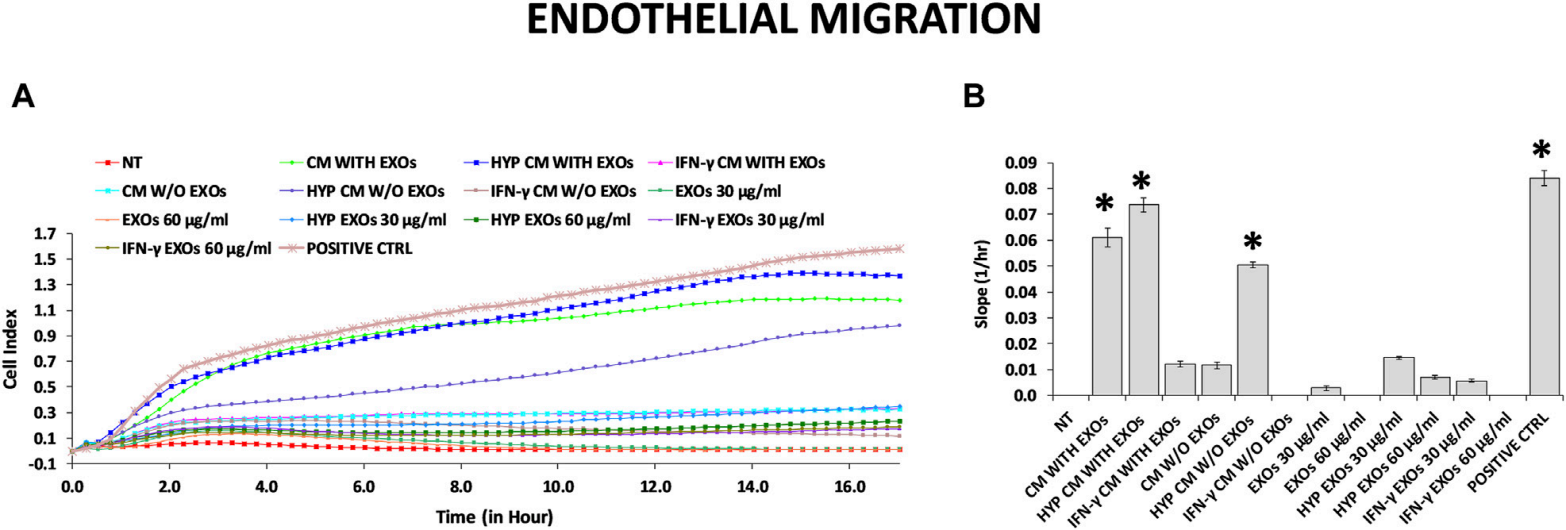
HUVEC migration assay. **(A)** Real-time migration monitoring of HUVECs with the xCELLigence system. **(B)** Slopes of migration curves. Untreated serum-free DMEM (NT); Untreated DMEM with serum (positive ctrl); Serum-free DMEM conditioned by hAMSCs (CM with EXOs); Serum-free DMEM conditioned by hAMSCs depleted of EXOs (CM w/o EXOs); Serum-free DMEM conditioned by hypoxic hAMSCs (HYP CM with EXOs); Serum-free DMEM conditioned by hypoxic hAMSCs depleted of EXOs (HYP CM w/o EXOs); Serum-free DMEM conditioned by IFN-γ-treated hAMSCs (IFN-γ CM with EXOs); Serum-free DMEM conditioned by IFN-γ-treated hAMSCs depleted of EXOs (IFN-γ CM w/o EXOs); 30 μg/mL EXOs secreted by hAMSCs (EXOs 30 μg/mL); 60 μg/mL EXOs secreted by hAMSCs (EXOs 60 μg/mL); 30 μg/mL EXOs secreted by hypoxic hAMSCs (HYP EXOs 30 μg/mL); 60 μg/mL EXOs secreted by hypoxic hAMSCs (HYP EXOs 60 μg/mL); 30 μg/mL EXOs secreted by IFN-γ-treated hAMSCs (IFN-γ EXOs 30 μg/mL); 60 μg/mL EXOs secreted by IFN-γ-treated hAMSCs (IFN-γ EXOs 60 μg/mL). Data are presented as the means ± SD of quadruplicate in two independent experiments. ∗*p* < 0.05 vs. NT.

The functional angiogenic effects of both CM and EXOs produced with or without priming were examined by analyzing *in vitro* two important aspects of the angiogenesis process: endothelial cell migration and the formation of capillary-like structures (tube formation). As expected, the highest amount of capillary structures was observed in the positive control group (DMEM with FBS, positive ctrl), and the smallest was observed in the negative control group (DMEM without FBS and treatments, NT). Compared with those on the NT, we found that HUVECs plated on basement membrane extracts (BMEs) were able to form capillary-like structures mainly when cultivated with complete CM from conventional hAMSC culture or CM and EXOs from hypoxic hAMSC cultures (Figures 4A,B). We quantified the differences across the treatments and found that all capillary parameters increased significantly in response to complete hypoxic CM (HYP CM) but not in response to HYP CM without EXOs (Figures 4C–H). Moreover, both the number of nodes and the tube length increased significantly with complete conventional CM (Figures 4C,H). Interestingly, treatment with hypoxic exosomes (HYP EXOs) at a concentration of 60 μg/mL also increased the amount of specific capillary parameters, such as the number of nodes, master segment length, and tube length (Figures 4C, F, H). Very few or no effects on capillary-like structure formation were observed when cells were treated with CM or EXOs derived from IFN-γ priming (Figure 4). Interestingly, we observed that the migration-related behavior of HUVECs was similar to that of tube formation. We observed a marked increase in HUVEC migration in the presence of conventional CM, HYP CM or HYP CM without EXOs (in contrast to the tube formation assay) (Figure 5).

### 3.4 Conditioned medium and exosomes produced through the priming of hAMSCs induce recruitment of neutrophils and activation of phagocytosis

**FIGURE 6:**
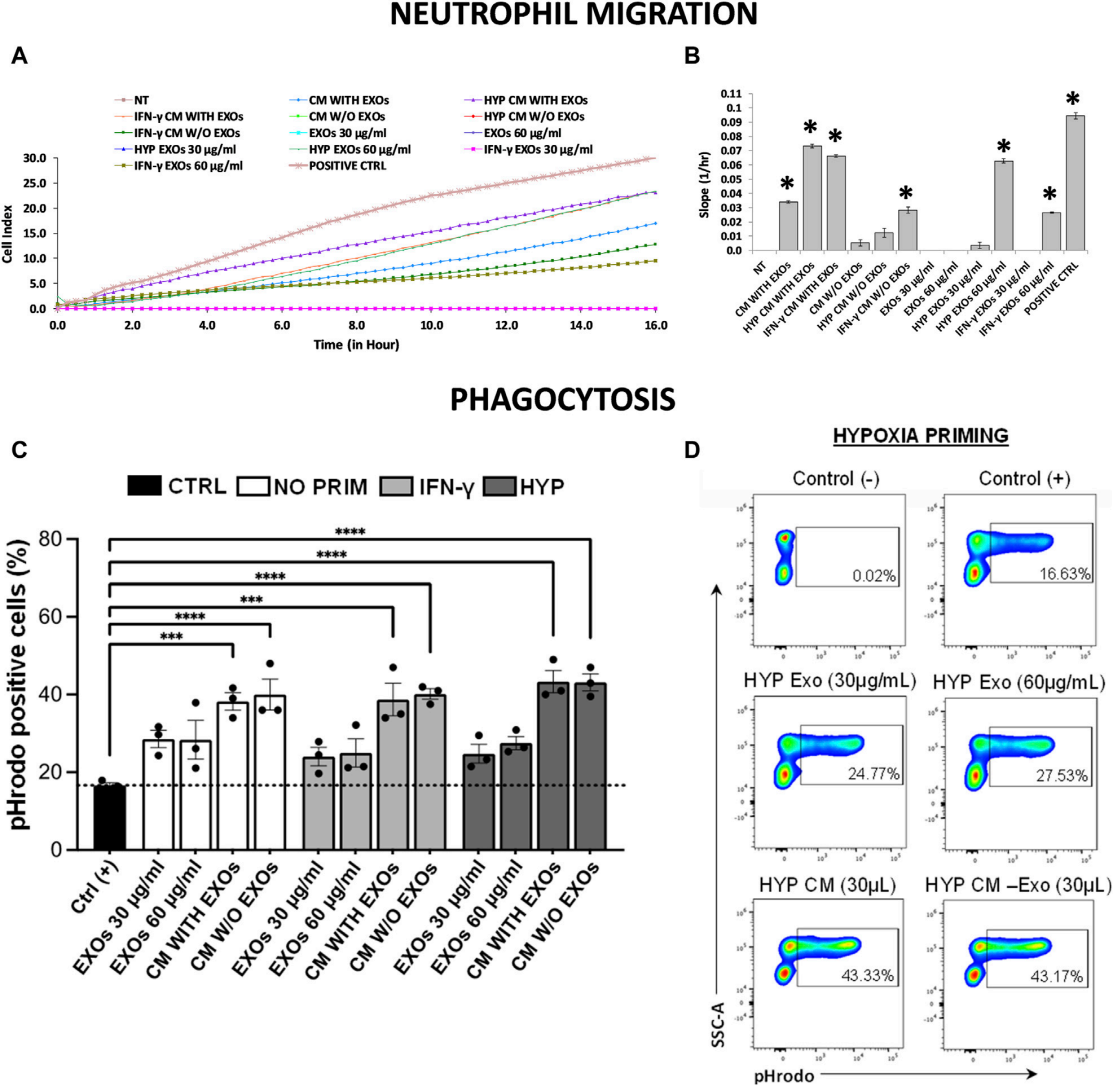
Neutrophil migration and phagocytosis assays. **(A)** Real-time migration monitoring of neutrophils with the xCELLigence system. **(B)** Slopes of migration curves. **(C, D)** Analysis of phagocytosis in blood samples using pHrodo-labeled particles. **(C)** The histogram shows the percentages of pHrodo + cells analyzed by flow cytometry after each treatment. **(D)** Representative plots showing the percentages of pHrodo-labeled cells after treatment of blood samples with both CM and EXOs derived from hypoxic hAMSCs. Untreated serum-free DMEM (NT); Untreated DMEM with serum (positive ctrl); Serum-free DMEM conditioned by hAMSCs (CM with EXOs); Serum-free DMEM conditioned by hAMSCs depleted of EXOs (CM w/o EXOs); Serum-free DMEM conditioned by hypoxic hAMSCs (HYP CM with EXOs); Serum-free DMEM conditioned by hypoxic hAMSCs depleted of EXOs (HYP CM w/o EXOs); Serum-free DMEM conditioned by IFN-γ-treated hAMSCs (IFN-γ CM with EXOs); Serum-free DMEM conditioned by IFN-γ-treated hAMSCs depleted of EXOs (IFN-γ CM w/o EXOs); 30 μg/mL EXOs secreted by hAMSCs (EXOs 30 μg/mL); 60 μg/mL EXOs secreted by hAMSCs (EXOs 60 μg/mL); 30 μg/mL EXOs secreted by hypoxic hAMSCs (HYP EXOs 30 μg/mL); 60 μg/mL EXOs secreted by hypoxic hAMSCs (HYP EXOs 60 μg/mL); 30 μg/mL EXOs secreted by IFN-γ-treated hAMSCs (IFN-γ EXOs 30 μg/mL); 60 μg/mL EXOs secreted by IFN-γ-treated hAMSCs (IFN-γ EXOs 60 μg/mL). Data are presented as the means ± SD of quadruplicate in a and b and triplicate in c and **(D)** ∗*p* < 0.05 vs. NT in **(B)**. ∗∗∗*p* < 0.001 and ∗∗∗∗*p* < 0.0001 vs. positive control in **(C)**.

Using a real-time transwell migration assay, we analyzed the ability of both CM and EXOs to recruit neutrophils. We observed that CM produced by both hypoxia and IFN-γ priming stimulates intense chemotaxis in neutrophils (approximately 2-fold increase) compared to CM produced by conventional hAMSCs, which induces moderate chemotaxis. Relevant chemotaxis was also induced by HYP EXOs at a concentration of 60 μg/mL (comparable to that of complete IFN-γ CM), IFN-γ CM without EXOs, and IFN-γ EXOs at a concentration of 60 μg/mL (comparable to that of complete conventional CM) (Figures 6A,B).

A phagocytosis assay was performed using peripheral blood from healthy adult volunteers. We preliminarily optimized the analysis in terms of time, dose and minimum blood volume requirement. As expected, nonphagocytic cells did not fluoresce (Figure 6D, negative control), whereas the positive control caused a significant increase in phagocytic cells (approximately 17%) (Figures 6C,D, positive control: blood samples exposed to pHrodo Green *E. coli* BioParticles). Interestingly, incubation of whole blood with pHrodo-labeled bacteria in the presence of CM, IFN-γ CM or HYP CM (with or without EXOs), resulted in a marked and significant shift in the fluorescence of phagocytic cells (approximately 39% for both CM and IFN-γ CM and 43% for HYP CM) compared to that of the positive control. Although we observed an increase in phagocytic cells after treatment with all types of EXOs at both 30 and 60 μg/mL (approximately 28%, 24%, and 25% with EXOs, IFN-γ EXOs and HYP EXOs, respectively), we did not find significant differences compared to the positive control (Figures 6C,D). The gating strategy for obtaining viable cells is shown in Supplementary Figure S1.

### 3.5 Dynamic analysis of immune responses to LPS in the presence or absence of hAMSC-derived CM or EXOs through simultaneous quantification of secreted cytokines in whole blood

**FIGURE 7:**
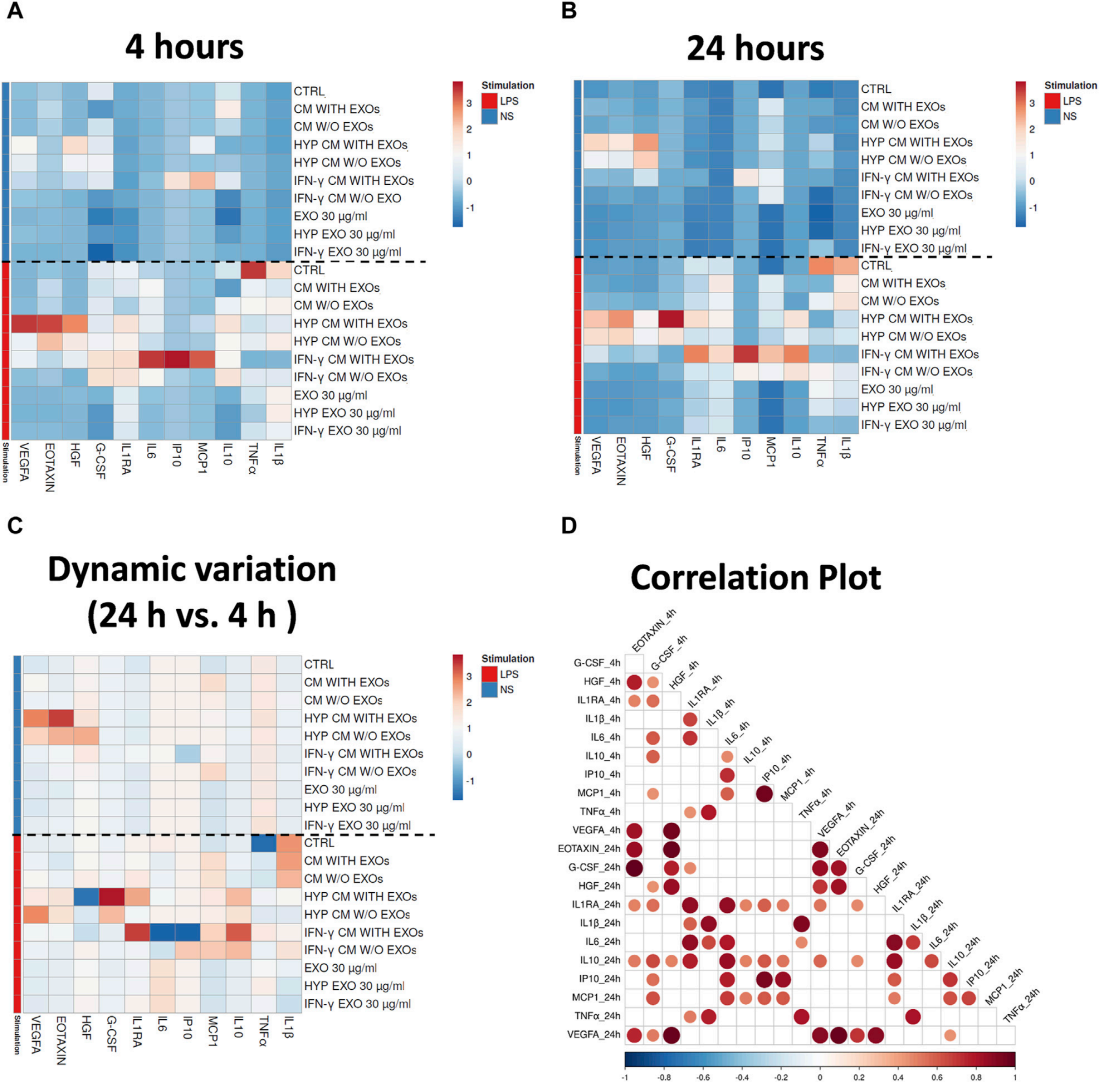
Secretion clusters (z-scores) of both up- and downregulated proteins in peripheral blood. Quantification of soluble factors secreted by peripheral blood cells stimulated or unstimulated with LPS in the presence or absence of each treatment after **(A)** four and **(B)** 24 h of culture. **(C)** Dynamic variations of factors between 4 and 24 h. **(D)** Correlation matrix of the factors. The degree of correlation between the two factors is shown through color intensity and the diameter of the circles. Significance was analyzed using a Spearman rank test, and the level of significance was set at *p* < 0.05. Whole blood without treatments (ctrl); Whole blood with serum-free DMEM conditioned by hAMSCs (CM with EXOs); Whole blood with serum-free DMEM conditioned by hAMSCs depleted of EXOs (CM w/o EXOs); Whole blood with serum-free DMEM conditioned by hypoxic hAMSCs (HYP CM with EXOs); Whole blood with serum-free DMEM conditioned by hypoxic hAMSCs depleted of EXOs (HYP CM w/o EXOs); Whole blood with serum-free DMEM conditioned by IFN-γ-treated hAMSCs (IFN-γ CM with EXOs); Whole blood with serum-free DMEM conditioned by IFN-γ-treated hAMSCs depleted of EXOs (IFN-γ CM w/o EXOs); Whole blood with 30 μg/mL EXOs secreted by hAMSCs (EXOs 30 μg/mL); Whole blood with 60 μg/mL EXOs secreted by hAMSCs (EXOs 60 μg/mL); Whole blood with 30 μg/mL EXOs secreted by hypoxic hAMSCs (HYP EXOs 30 μg/mL); Whole blood with 60 μg/mL EXOs secreted by hypoxic hAMSCs (HYP EXOs 60 μg/mL); Whole blood with 30 μg/mL EXOs secreted by IFN-γ-treated hAMSCs (IFN-γ EXOs 30 μg/mL); Whole blood with 60 μg/mL EXOs secreted by IFN-γ-treated hAMSCs (IFN-γ EXOs 60 μg/mL).

We evaluated the immune functional effects of both primed and unprimed hAMSC-derived products through a sequential analysis (at both 4 and 24 h) of the secretion of different factors in whole blood stimulated with LPS. We analyzed angiogenic factors as well as pro- and anti-inflammatory factors, such as tumor necrosis factor alpha (TNFα), interleukin 1 beta (IL1β), CCL11 (Eotaxin), hepatocyte growth factor (HGF), vascular endothelial growth factor A (VEGFA), CXCL10 (IP10), CCL2 (MCP1), interleukin 1 receptor antagonist (IL1RA), interleukin 6 (IL6), colony stimulating factor 3 (G-CSF), and IL10, and observed different expression patterns when blood was stimulated or not with LPS for both 4 and 24 h (Figures 7A,B). Treatment with LPS effectively induced an increase in pro-inflammatory cytokines such as TNFα and IL1β in control samples (whole blood without hAMSC-derived CM or EXOs stimulated with LPS, ctrl) after both 4 and 24 h, whereas a reduction in the same cytokines was observed in the presence of both primed and unprimed hAMSC-derived CM and EXOs (Figures 7A,B). Interestingly, after both 4 and 24 h, a significant increase in crucial angiogenic factors such as Eotaxin and VEGFA was observed in response to HYP CM treatment, and a slightly less intense effect was also observed in response to HYP CM w/o EXOs. Additionally, in the same treatment groups, the production of the pro-angiogenic factor HGF was higher only at 4 h (Figure 7A). In contrast, 4 h of treatment with IFN-γ CM increased the production of crucial immunomodulatory factors, such as IP10, MCP1, IL1RA, IL6, and G-CSF, whereas the production of only IP10, MCP1, IL1RA, and IL6 increased after 24 h of treatment. Weak effects at both 4 and 24 h were observed following treatment with IFN-γ CM w/o EXOs. Notably, treatment with IFN-γ CM for 24 h induced a significant increase in IL10 production (Figure 7B). Furthermore, although treatment with all types of EXOs inhibited the overproduction of pro-inflammatory cytokines, such as TNFα and IL1β, it did not affect the production of any of the other factors analyzed (Figures 7A,B). To investigate the dynamic variations in the aforementioned functional factors, we also evaluated the differences in concentrations between 4 and 24 h. As shown in Figure 7C, significant increases in Eotaxin and VEGFA were detected with HYP CM treatment (with or without EXOs), whereas no significant variation was observed for HGF. The same treatment also induced a variation in the production of IL1RA, G-CSF and IL10. Moreover, treatment with IFN-γ CM induced a significant overproduction of IL1RA, IL10 and MCP1. Using correlation analysis, we found that the pro-angiogenic factor VEGFA significantly correlated with other pro-angiogenic factors, such as Eotaxin and HGF, after both 4 and 24 h of treatment. Significant correlations were also observed among the immunomodulatory factors after both 4 and 24 h of treatment (Figure 7D).

## 4 Discussion

MSCs exhibit robust immunoregulatory, angiogenic and regenerative characteristics (Iijima et al., 2018; Miceli et al., 2019; Pittenger et al., 2019; Bulati et al., 2020; Lo Nigro et al., 2021; Bulati et al., 2023). Consequently, they have been investigated extensively in the field of regenerative medicine for the treatment of various diseases (Cittadini et al., 2022; Miceli and Bertani, 2022; Miceli et al., 2023a; Russo et al., 2023b). Recent scientific findings have elucidated that products derived from MSCs, such as CM and EXOs, may contribute, at least partially, to the therapeutic effects of MSCs (Miceli et al., 2021a; Chinnici et al., 2021; Takeuchi et al., 2021; Alberti et al., 2022; Russo et al., 2023a). Intriguingly, diverse priming strategies can enhance the therapeutic properties of both MSCs and their derived products (Noronha et al., 2019; Miceli et al., 2021b; Gallo et al., 2022; Miceli et al., 2023b).

Our study explored the impact of distinct priming strategies, specifically hypoxia and IFN-γ treatment, on the proteomic profile of the hAMSC-derived secretome. We used amnion-derived MSCs for their numerous advantages, including their abundance, non-invasive procurement, and ease of cultivation to a transplantable quantity, thereby avoiding ethical concerns associated with allografting (Parolini et al., 2009). Our findings indicate that both hypoxia and IFN-γ priming effectively enhanced the paracrine regenerative properties of hAMSC-derived CM and EXOs by promoting the production of functional factors associated with angiogenesis, immune system regulation, and tissue regeneration (Figures 2, 3). In particular, as revealed by our GO analysis, we observed that biological processes related to the regulation of angiogenesis were mainly targeted by CM and EXOs derived from hAMSCs primed with hypoxia. Hypoxic hAMSCs were also capable of producing functional factors that activate specific processes, such as neutrophil/macrophage activation, leukocyte chemotaxis and phagocytosis, ultimately activating innate immune responses. On the other hand, treatment with IFN-γ induced hAMSCs inhibited immune system activation by stimulating processes such as negative regulation of IFN-γ production, negative regulation of the immune effector process and negative regulation of the immune response (Figures 2F–I and 3F-I). Our data revealed that hypoxic priming induced an increase in the production of crucial angiogenic factors, including VEGFA and angiopoietin-like 4 (ANGPTL4), in both CM and EXOs and the in the production of endoglin (ENG) and platelet-derived growth factor receptor beta (PDGFRB), but only in EXOs (Supplementary Tables S3, S4). VEGFA, PDGFRB, ANGPTL4 and ENG have been shown to play major roles in angiogenesis and vascular homeostasis, not only in physiological regeneration but also in most pathological angiogenic processes such as cancer (Raica and Cimpean, 2010; Nassiri et al., 2011; Shibuya, 2011; Uccelli et al., 2019; Fernandez-Hernando and Suarez, 2020). Additionally, hypoxic hAMSCs produced functional factors such as CXCL3 (GRO-γ), CXCL8 (IL8) and CXCL1 (GRO-α) (Supplementary Tables S3, S4), which play roles in angiogenesis as well as in chemotaxis/activation of crucial cell components of the innate immune system, such as neutrophils and macrophages (Lukaszewicz-Zajac et al., 2020; Sokulsky et al., 2020; Cambier et al., 2023). In contrast, IFN-γ priming led to the overproduction of immunosuppressive factors, such as transforming growth factor beta 1 (TGFB1) and annexin A1 (ANXA1), both in CM and EXOs; thrombospondin 1 (THBS1), only in EXOs; and homer scaffold protein 2 (HOMER2), granulin precursor (GRN), toll interacting protein (TOLLIP) and CCL2 (MCP-1), only in CM (Supplementary Tables S3, S4). TGFB1 and ANXA1 have been shown to be very effective at limiting inflammation in several experimental models (Gavins and Hickey, 2012; Sanjabi et al., 2017). THBS1 has immunosuppressive effects that regulate the function/activation of multiple immune cells (Kaur and Roberts, 2024). HOMER2 can negatively regulate both IL-2 expression and T cell activation through competition with calcineurin and through binding with nuclear factor of activated T cells (NFAT) (Huang et al., 2008). In various immune-related diseases, the GRN protein has been shown to have anti-inflammatory effects by inhibiting TNFα activity (Tian et al., 2014). TOLLIP has been implicated in the control of inflammatory cytokine production and is crucial as a negative regulator of IL-1-activated NF-κB signaling (Didierlaurent et al., 2006). MCP-1 is a monocyte chemoattractant that can have immunosuppressive effects inducing the generation of immunoregulatory dendritic cells (DCreg) (Kudo-Saito et al., 2013). Therefore, our data showed that the composition of functional factors in CM and EXOs varies with the specific priming strategy, leading to distinct functional responses. Hypoxia priming appears to polarize naïve hAMSCs to stimulate angiogenesis, and activate innate immune responses and tissue regeneration. In contrast, IFN-γ treatment induces an anti-inflammatory and pro-trophic phenotype in hAMSCs, regulating inflammatory responses and facilitating tissue remodeling.

In accordance with the observed proteomic profiles, we confirmed the greater angiogenic properties of the hypoxic hAMSC products through *in vitro* functional assays. In particular, hypoxia-induced CM significantly enhanced the formation of capillary-like structures and the migration of endothelial cells (Figures 4, 5). Notably, recruitment of endothelial cells has been shown to be essential for vascular growth (Carmeliet and Jain, 2011). Moreover, we demonstrated that both CM and EXOs derived from hypoxic priming conditions might also induce both neutrophil recruitment (Figures 6A,B) and phagocytosis (Figures 6C,D), crucial processes during the activation of innate immune responses (Selders et al., 2017).

Interestingly, our study goes beyond the direct effects of priming on hAMSCs, demonstrating that hypoxia and IFN-γ can influence the functional characteristics of the hAMSC-derived secretome, which, in turn, orchestrates the production of functional factors by peripheral blood cells (PBCs). In fact, we performed a whole blood assay in which the CM produced through distinct priming stimuli elicited the production of different functional factors by PBCs (Figure 7). Indeed, our data showed that while hypoxia priming induced the production and release of angiogenic factors such as VEGFA, HGF and EOTAXIN (Salcedo et al., 2001; Sulpice et al., 2009), IFN-γ priming was shown to stimulate the production of anti-inflammatory factors such as IP10, MCP1, the interleukin 1 receptor antagonist (IL1RA), IL6, G-CSF and IL10 (Grutz, 2005; Martins et al., 2010; Gupta et al., 2011; Hunter and Jones, 2015; Gschwandtner et al., 2019; Fan et al., 2023). This experiment also revealed that the treatment of PBCs with CM and EXOs primed with both hypoxia and IFN-γ inhibited the production of pro-inflammatory factors such as IL1β and TNFα (Ott et al., 2007) induced by LPS stimulation of PBCs (Figure 7). Notably, treatment with IFN-γ CM was able to stimulate IL10 production after 24 h of treatment (Figure 7B). IL10 is a crucial anti-inflammatory cytokine capable of inhibiting the production of both IL1β and TNFα (Cassatella et al., 1993). In a mouse model of septic shock, IL10 was shown to inhibit the *in vivo* production of TNFα protecting it from mortality (Gerard et al., 1993). In addition, considering the functional role of IL10 in the progression of inflammation (Iyer and Cheng, 2012), our findings revealed an important time-dependent effect of IFN-γ CM in regulating the resolution of inflammation.

**FIGURE 8:**
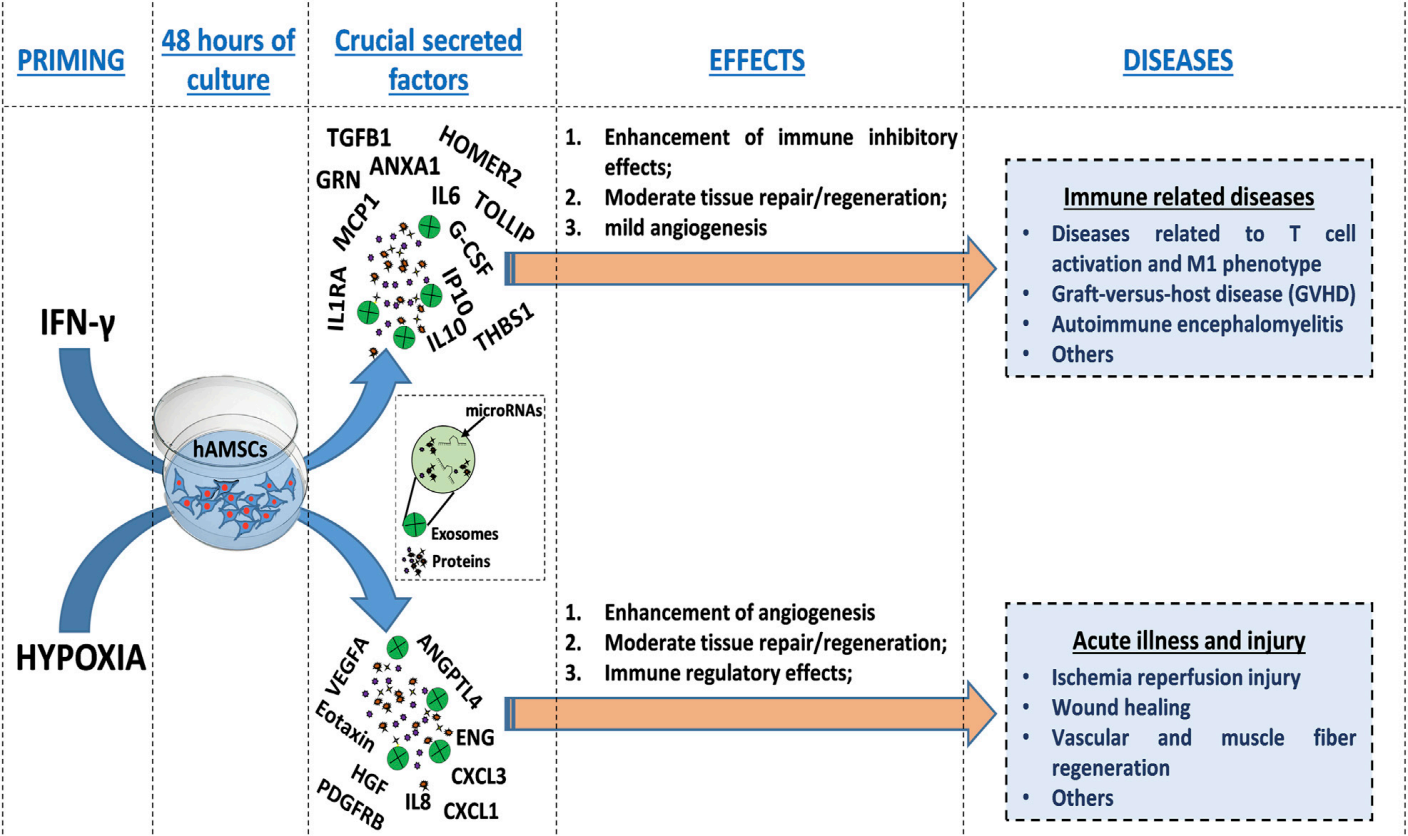
Schematic representation of the molecular effects and therapeutic potential of hAMSCs after priming. HAMSCs were preconditioned through hypoxia or IFN-γ treatment and each priming strategy differentially induced the production of specific factors (soluble factors or EXO-containing factors), which affected the activation of biological processes involved in angiogenesis, tissue repair/regeneration and the modulation of inflammation. Additionally, the priming strategies mentioned above also led to the preconditioning of hAMSCs, which in turn influenced the production of functional factors by peripheral blood cells (as shown in the whole blood assay results). Different priming strategies might be used to direct the therapeutic effects of hAMSCs toward specific diseases.

Our comprehensive protein characterization and functional analyses collectively indicate that hypoxia-primed hAMSCs exhibited a greater propensity to stimulate angiogenesis, while IFN-γ priming demonstrated a heightened capacity to induce immunosuppressive effects. As illustrated in Figure 6, both priming strategies also effectively stimulate innate immune response activation. The paracrine effects observed in this study seem to be mostly linked to the presence of the CM and not to the presence of EXOs, as suggested by the minimal effects observed upon EXO treatment in the tube formation assay and neutrophil migration (Figures 4, 5). Additionally, no significant differences were detected between whole CM and EXO-depleted CM, emphasizing the pivotal role of CM in mediating the paracrine effects of hAMSCs (Figure 5; 7). Based on our findings, we speculate that angiogenesis promotion induced by hypoxia-primed hAMSCs is a promising strategy for addressing pathological conditions characterized by inadequate or abnormal vessel formation. This potential role can also be applied to the wound healing process (Tonnesen et al., 2000), vascular growth during tissue regeneration (Saberianpour et al., 2018), and the context of ischemia (Hayashi et al., 2006). Furthermore, the activation of innate immune responses by hypoxic hAMSCs may play a crucial role in orchestrating the resolution of such pathologies (Julier et al., 2017). In contrast, the use of hAMSCs primed with IFN-γ, which has major immunosuppressive effects, might be therapeutically useful for the treatment of diseases characterized by an exacerbation of immune system activity (Chen and Brosnan, 2006; Leite et al., 2021; Kadri et al., 2023) (Figure 8).

In this study, we highlight a potential way to optimize MSC-based therapies, with the overall goal of improving their efficacy and mitigating the suboptimal outcomes observed in numerous clinical trials (Squillaro et al., 2016; Lukomska et al., 2019; Fricova et al., 2020; Zhou et al., 2021). As demonstrated in physiological contexts, MSCs undergo functional activation during stress conditions such as hypoxia and inflammatory environments (Miceli et al., 2021b; Miceli et al., 2023b). Consequently, employing priming strategies may prove decisive in directing MSC therapeutic properties toward specific disease classes (Figure 8) and in facilitating the use of MSC-derived products, such as CM and EXOs, rather than the cells themselves (Miceli et al., 2021b; Miceli et al., 2023b). Notably, the utilization of hAMSC products presents numerous advantages for clinical translation, encompassing considerations related to manufacturing, logistics, safety, and regulatory aspects.

Another important aspect highlighted in this study concerns EXOs. In the last decade, the role of MSC-derived EXOs in regulating inflammatory responses and tissue regeneration remained unclear. However, these functional vesicles have demonstrated similar beneficial effects to their parent cells in suppressing various autoreactive immune cells or inducing cutaneous wound healing (Baharlooi et al., 2020; Hu et al., 2022). Furthermore, there is the possibility to enhance EXO-therapeutic potential by tissue-engineered MSCs or by direct engineering of EXOs. In these cases, specific molecules and/or receptors can enable EXOs to target specific pathways of interest (Baharlooi et al., 2020; Hu et al., 2022; Raghav et al., 2022). In line with that, although our study emphasizes that the functional effects of MSCs are primarily associated with the use of CM, our data reveal that the EXO protein composition is also influenced by priming. It is well established that EXOs, to some extent, play a role in mediating the therapeutic effects of MSCs (Alberti et al., 2022; Russo et al., 2023a; Bulati et al., 2023). Therefore, further studies are needed to determine the role of EXOs in mediating the paracrine effects of MSCs, and how priming might be used to improve their therapeutic properties.

## 5 Conclusion

Our data suggest that distinct priming strategies significantly alter the protein composition of hAMSC-derived CM and EXOs leading to enhanced therapeutic effects. Hypoxic priming emphasizes angiogenesis, while IFN-γ priming has immunosuppressive effects. We have confirmed these results through functional studies and reveal the potential of fine-tuning the therapeutic properties of hAMSCs in a priming-dependent manner. This strategy provides new perspectives for enhancing the therapeutic efficacy of MSCs and guiding their therapeutic effects towards targeted pathologies. These cells are able to release a plethora of regulatory bioactive factors that, if opportunely modulated by specific priming strategies, might be capable of acting simultaneously on multiple targets and sustaining the therapeutic effects of MSCs, mainly in certain so-called multifactorial diseases (for which multiple molecular targets are involved in the pathogenesis), including Alzheimer’s, Parkinson’s disease (Youdim et al., 2014; Kumar et al., 2022), cancer (Petrelli and Giordano, 2008) and ischemia-reperfusion injury (Davidson et al., 2019). These insights offer ways to optimize MSC-based therapies, potentially improving their effectiveness and addressing challenges faced in clinical trials. Our study underscores the potential of hAMSC-derived products, particularly CM, in clinical translations, considering the numerous advantages that this approach entails. Moreover, our findings pave the way for further studies to understand the role of EXOs in mediating MSC paracrine effects and optimize their therapeutic use in multifactorial pathologies. This study highlights that MSC-based therapeutic products could be effective therapies for several diseases in the field of regenerative medicine.

While our study provides valuable insights into the impact of distinct priming strategies on the therapeutic properties of MSCs, there are several limitations that should be acknowledged. Our research primarily relies on *in vitro* experiments to assess the effects of hypoxia and IFN-γ priming on hAMSC-derived secretome. While these experiments offer controlled environments to study cellular responses, they may not fully replicate the complexities of *in vivo* conditions, potentially limiting the translational relevance of our findings. Moreover, we focused exclusively on placenta as a cell source for our study for its advantages. However, this narrow focus limits the generalizability of our findings, as different cell sources may respond differently to priming strategies. Future studies should explore the effects of priming on MSCs derived from other tissues to provide a more comprehensive understanding.

## Figures and Tables

**Table:** 

**ANXA1**	Annexin A1
**AGC**	Automatic gain control
**ANGPTL4**	Angiopoietin-like 4
**BMEs**	Basement membrane extracts
**CI**	Cell index
**CM**	Conditioned medium
**DCreg**	Dendritic cells
**DDA**	Data-dependent acquisition
**ENG**	Endoglin
**EVs**	Extracellular vesicles
**EXOs**	Exosomes
**FASP**	Filter aided sample preparation
**FBS**	Fetal bovine serum
**GO**	Gene ontology
**G-CSF**	Colony stimulating factor 3
**GRN**	Granulin precursor
**hAMSCs**	Human amnion-derived mesenchymal stromal/stem cells
**HGF**	Hepatocyte growth factor
**HUVECs**	Human umbilical vein endothelial cells
**IDO**	Indoleamine 2,3-dioxygenase 1
**IL1β**	Interleukin 1 beta
**IL6**	Interleukin 6
**IL10**	Interleukin 10
**IL1RA**	Interleukin 1 receptor antagonist
**ISCT**	International Society for Cell and Gene Therapy
**LFQ**	Label-free quantification
**MSCs**	Mesenchymal stromal/stem cells
**NCE**	Normalized collision energy
**NFAT**	Nuclear factor of activated T cells
**NTA**	Nanoparticle tracking analysis
**PBCs**	Peripheral blood cells
**PBS**	Phosphate-buffered saline
**PGE2**	Prostaglandin E synthase 2
**PCA**	Principal component analysis
**SD**	Standard deviation
**STAGE**	Stop-and-go extraction
**TGFB1**	Transforming growth factor beta 1
**THBS1**	Thrombospondin 1
**TNFα**	Tumor necrosis factor alpha
**TOLLIP**	Toll interacting protein
**VEGFA**	Vascular endothelial growth factor A

## Data Availability

The data presented in the study are deposited in the ProteomeXchange repository accession number PDX052445, via the link http://www.ebi.ac.uk/pride/archive/projects/PXD052445.
