# Supplementary figures and images for: Proteomic analysis and functional validation reveal distinct therapeutic capabilities related to priming of mesenchymal stromal/stem cells with IFN-γ and hypoxia: potential implications for their clinical use
Source: Front Cell Dev Biol. 2024 May 31;12:1385712. doi: 10.3389/fcell.2024.1385712 (PMC11179434; doi:10.3389/fcell.2024.1385712)

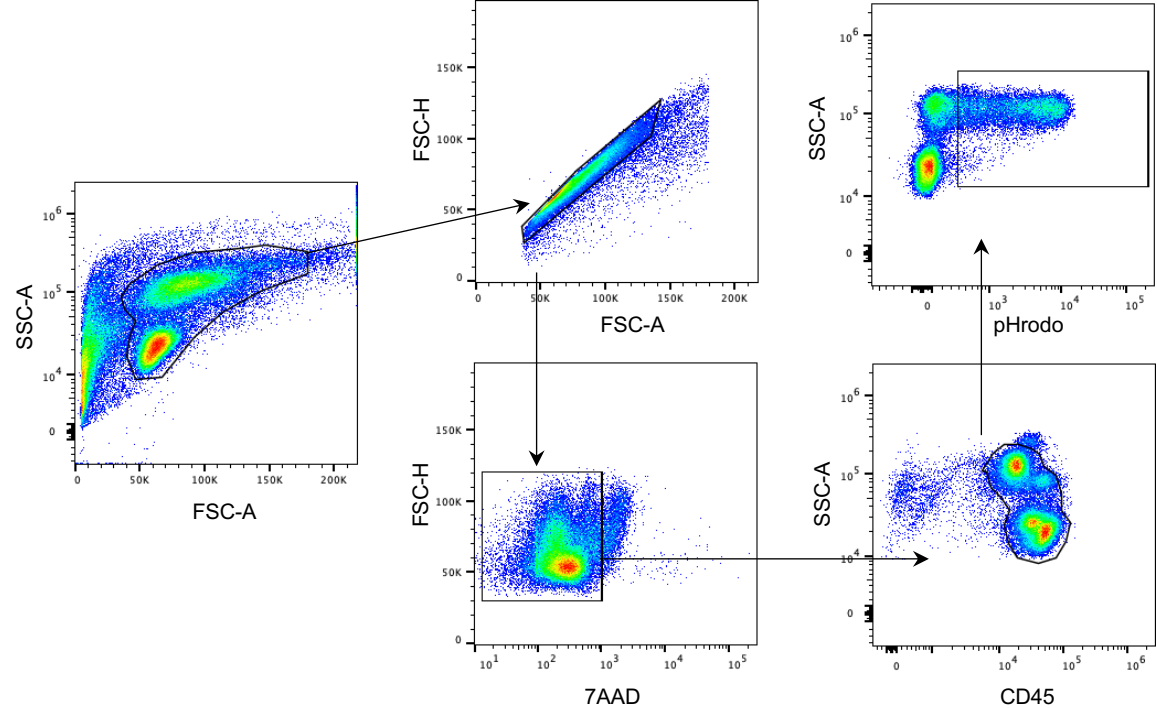

Supplement: Supplementary file 3 [file DataSheet1.PDF]
